# Therapeutic Dilemma of Natural Watchman: Congenital Absence of the Left Atrial Appendage

**DOI:** 10.1155/2018/7573425

**Published:** 2018-07-25

**Authors:** Phoo Pwint Nandar, Asim Kichloo, Thein Tun Aung, Kevin D. Kravitz

**Affiliations:** ^1^Department of Internal Medicine, Central Michigan University, Saginaw, MI, USA; ^2^Department of Electrophysiology, University of Iowa, Iowa City, IA, USA; ^3^Department of Electrophysiology, Good Samaritan Hospital, Dayton, OH, USA

## Abstract

Congenital absence of the left atrial appendage is a rare congenital cardiac anomaly which is usually an incidental finding. We present a rare case of congenital absence of the left atrial appendage in a 77-year-old female patient with atrial fibrillation, and we will discuss the role of anticoagulation in the patient with congenital absence of the left atrial appendage based on the scientific data and theoretic background.

## 1. Introduction

Congenital absence of the left atrial appendage (CALAA) is an extremely rare congenital cardiac anomaly. Given the fact that it is very rare, its clinical significance and the chance of developing intracardiac thrombi are largely unknown. There are only a handful case reports noted in the medical literature, and none of them reported intracardiac thrombi. The anticoagulation therapy in patients with CALAA remains unclear.

## 2. Case Presentation

A 77-year-old female with hypertension, hyperlipidemia, and paroxysmal atrial fibrillation (AF) presented with palpitations. She has neither prior cardiovascular procedure nor cardiac surgery. AF was diagnosed 4 years ago, and she was started on oral anticoagulation with warfarin. She was active and independent. She was very symptomatic with frequent palpitations. Dofetilide was started for rhythm control. She was successfully cardioverted in January 2017 while on dofetilide and warfarin. However, she went back to AF in March 2017. Dofetilide dose was adjusted. Then, she converted to sinus rhythm and always reported to be compliant with the medication. In May 2017, she had palpitations and was found to be in AF again. Given the fact that symptomatic AF is affecting her daily life and she failed rhythm control therapy, ablation was offered. She underwent cryoablation and pulmonary vein isolation procedure.

**Figure 1:**
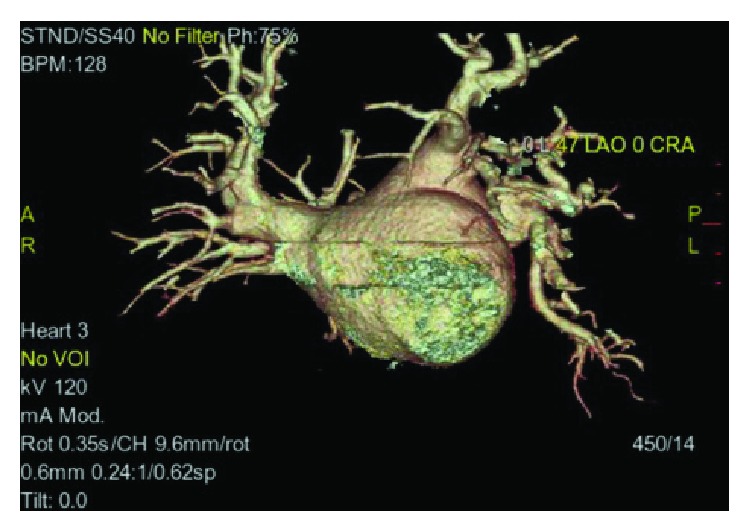
Computed tomography angiogram (CTA) of the pulmonary veins at 47 degrees left anterior oblique (LAO) view showing there is no left atrial appendage on the left atrium.

**Figure 2:**
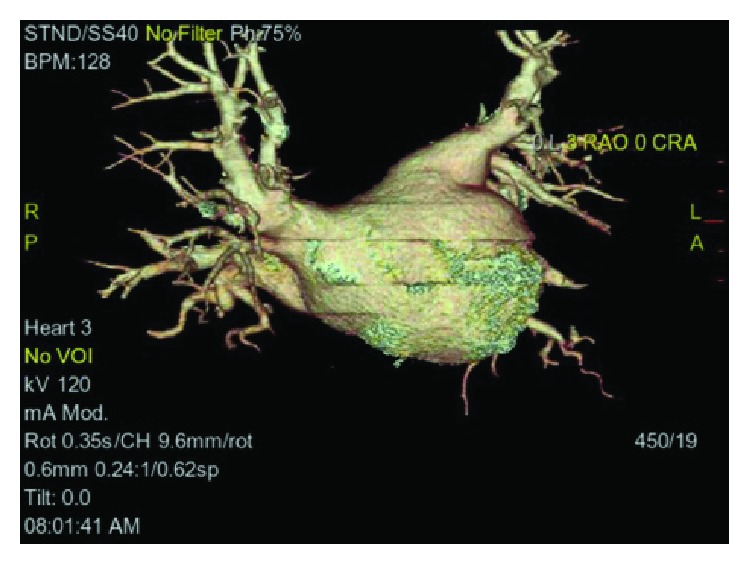
CTA of the pulmonary veins at anteroposterior (AP) view showing there is no left atrial appendage.

**Figure 3:**
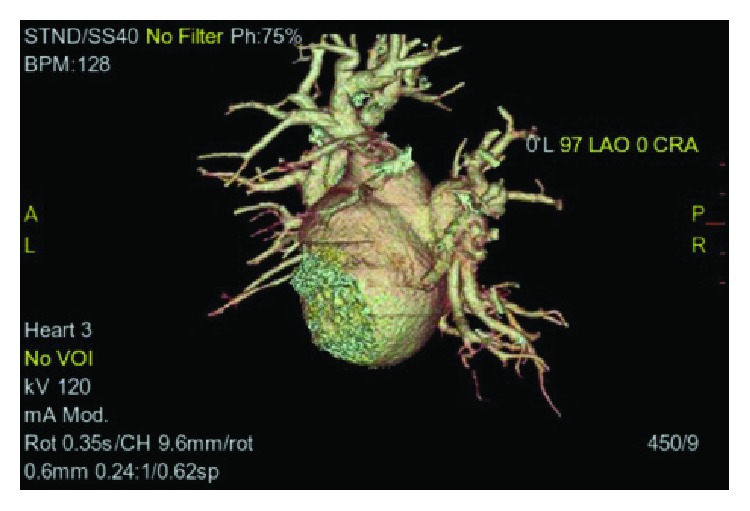
CTA of the pulmonary veins at 97 degrees steep LAO view showing there is no left atrial appendage on the heart surface.

**Figure 4:**
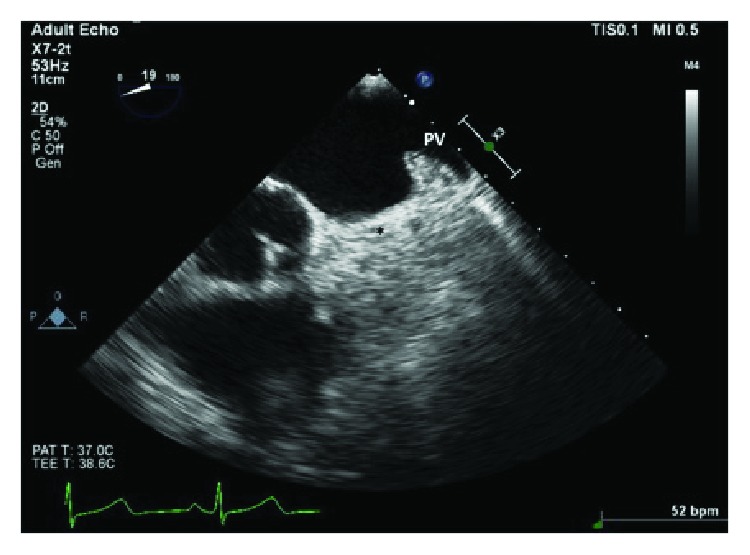
Transesophageal echocardiogram (TEE) at aortic valve level, 19-degree angle midesophageal view shows the pulmonary vein but the absence of the left atrial appendage at the usual anatomical location marked with an asterisk (^∗^).

**Figure 5:**
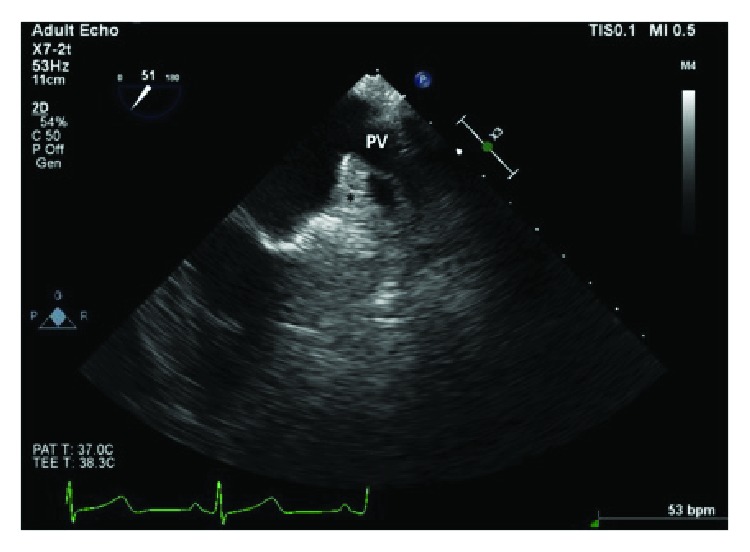
TEE at 50 degrees angle midesophageal view showing the coumadin ridge and pulmonary vein (PV). Note that there is no left atrial appendage in the area marked with (^∗^).

Computed tomography angiogram of the pulmonary veins (Figures 1–3) and transesophageal echocardiogram (Figures 4 and 5) were ordered prior to the procedure as evaluations for the cardiac anatomy and to rule out intracardiac thrombi, which revealed she had no left atrial appendage. Anticoagulation therapy with warfarin was continued as per current guidelines since we have no data on anticoagulation management in CALAA.

## 3. Discussion

LAA is a small muscular extension abutting from the upper part of the left atrium. Embryologically, the LAA develops as early as the third week of embryonic life [1]. The LAA produces a high level of atrial natriuretic factor and contributes to the contractile function of the left atrium [2]. In adults, it is described as a narrow, tubular, single or multilobed structure functioning as a decompression chamber during left ventricular systole and other periods of high left atrial pressure [1].

The major clinical significance of the LAA is not in the functionality but for its lethality. In the past, the left atrial appendage (LAA) has been considered to be a relatively insignificant portion of cardiac anatomy. It is now recognized that it is a structure with important pathological associations [1]. The LAA is the only area within the left atrium that is composed of pectinate muscle and creates a milieu that is conducive to blood stasis and thrombus formation. It is estimated that up to 90% of AF-related thrombi are located in the LAA [3]. Approximately 87% of all strokes are ischemic infarctions [4]. Cardiogenic embolism is responsible for 14–30% of all ischemic strokes [5]. A multicenter study has found that patients with the chicken wing morphology are significantly less likely to have an embolic event compared to those with cactus, windsock, and cauliflower morphologies [6].

Evaluation of the LAA by cardiac imaging studies prior to intracardiac procedures is usually done to rule out intracardiac thrombi. Reduced or absent LAA inflow and outflow velocities and low LAA ejection fractions are associated with LAA thrombus formation [1]. Oral anticoagulants are the current standard of care in preventing CVA in patients with AF. However, all anticoagulants such as warfarin, dabigatran, rivaroxaban, apixaban, and edoxaban carry the risk of bleeding. They are contraindicated in patients with prior hemorrhagic strokes and untreated bleeding disorders. Obliterating the LAA with the device (Watchman device) has been recently approved as an alternative option for patients who cannot tolerate long-term anticoagulation therapy. The remarkably successful Watchman trials have demonstrated that the Watchman device competes favorably with warfarin in terms of stroke and cardiovascular mortality [7].

Advanced sonographic techniques such as biplane and multiplane TEE have enabled physicians to visualize the LAA in most cases [8]. Nonvisualization of the LAA during TTE can be due to either of the following causes: (A) fresh thrombus, (B) variant anatomical features, (C) poor echocardiographic window, (D) prior surgical ligation, (E) presence of an implanted occluder device, or (F) CALAA [2]. For further differentiation, detailed history taking, looking at prior surgical records, and highly sophisticated imaging modalities like multidetector CT and cardiac MRI would be of immense help [8]. Clamping of the LAA during cardiac surgery results in an increase in left atrial pressure and dimension as well as in transmitral and pulmonary diastolic flow velocities [9]. Because of its increased distensibility, the LAA may augment hemodynamic function by modulating left atrial pressure-volume relations in the states of increased left atrial pressure and volume overload [10].

Theoretically, the risk of cerebrovascular accident (CVA) in CALAA should be lower since it removes the anatomical source of cardiac embolism [11]. However, there is no strong scientific data suggesting a favorable prognosis of CALAA and lower incidence of left atrium thrombus formation. The clinical implication for CALAA with AF in terms of anticoagulation even after ablation procedures still remains a mystery [12]. Given the fact of a very low incidence of CALAA, it is not likely to have a randomized trial on anticoagulation therapy.

According to the 2017 expert consensus statement on ablation of AF, the decision to discontinue anticoagulation after ablation should be based on the patient's stroke risk profile, not on the clinical outcome of the procedure [13]. Therefore, in the case we presented, anticoagulation was continued even after a successful ablation because her CHA_2_DS_2_-VASc score was >2, and there is no proven data on the safety of discontinuation of anticoagulation in CALAA.

## 4. Conclusion

CALAA is a very rare anomaly, which can change anticoagulation management strategies in a patient with AF, and there is very limited data to confirm the lower risk of stroke in a patient with CALAA. Given the importance of the LAA in the clot formation and embolization causing CVA in an AF patient, whether its congenital absence decreases the risk of thrombus formation is yet to be seen.
